# Prognostic Significance of Modified Advanced Lung Cancer Inflammation Index in Patients With Renal Cell Carcinoma Undergoing Laparoscopic Nephrectomy: A Multi-Institutional, Propensity Score Matching Cohort Study

**DOI:** 10.3389/fnut.2021.781647

**Published:** 2022-01-20

**Authors:** Weipu Mao, Keyi Wang, Yuan Wu, Jinliang Ni, Houliang Zhang, Yidi Wang, Zonglin Wu, Ruiji Liu, Jiang Geng, Shuqiu Chen, Ming Chen

**Affiliations:** ^1^Department of Urology, Shidong Hospital of Yangpu District, Shanghai, China; ^2^Department of Urology, Affiliated Zhongda Hospital of Southeast University, Nanjing, China; ^3^Department of Urology, Hefei Cancer Hospital, Chinese Academy of Sciences, Hefei, China; ^4^Department of Urology, School of Medicine, Shanghai Tenth People's Hospital, Tongji University, Shanghai, China

**Keywords:** renal cell carcinoma, advanced lung cancer inflammatory index, modified advanced lung cancer inflammatory index, overall survival, biomarker

## Abstract

**Background:**

We conducted a multi-institutional clinical study to assess the prognostic value of the advanced lung cancer inflammatory index (ALI) and modified ALI (mALI) in patients with renal cell carcinoma (RCC).

**Methods:**

We collected 440 patients who underwent laparoscopic nephrectomy at three centers from 2014 to 2019. ALI was defined as body mass index (BMI) × serum albumin (ALB)/neutrophil-to-lymphocyte ratio (NLR) and mALI as L3 muscle index × ALB/NLR. Kaplan-Meier curves, receiver operating characteristic (ROC) curves and Cox survival analysis were used to assess the effect of ALI and mALI on overall survival (OS). In addition, we performed 1:1 propensity score matching (PSM) for the high mALI and low mALI groups to further explore the impact of mALI on survival in RCC patients.

**Results:**

The optimal cut-off values for ALI and mALI were 40.6 and 83.0, respectively. Based on the cut-off values, we divided the patients into high ALI and low ALI groups, high mALI and low mALI groups. ALI and mALI were significantly associated with the AJCC stage, Fuhrman grade, T stage, and M stage. Low ALI (*p* = 0.002) or low mALI (*p* < 0.001) was associated with poorer prognosis. ROC curves showed that mALI was a better predictor of OS than ALI. Multivariate Cox regression analysis showed that low mALI (aHR = 2.22; 95% CI 1.19–4.13, *p* = 0.012) was an independent risk factor for OS in RCC patients who underwent nephrectomy, while ALI (aHR = 1.40; 95% CI 0.73–2.66, *p* = 0.309) was not significantly associated. Furthermore, after PSM analysis, we found that mALI remained an independent risk factor for OS (aHR = 2.88; 95% CI 1.33–6.26, *p* = 0.007) in patients with RCC.

**Conclusions:**

For RCC patients undergoing laparoscopic nephrectomy, low ALI and low mALI were associated with poor prognosis, and preoperative mALI can be used as a potential independent prognostic indicator for RCC patients.

## Introduction

Renal cancer, also known as renal cell carcinoma (RCC), is a malignant tumor originating from the urinary tubular epithelium of the renal parenchyma, and its incidence accounts for 2.2% of adult malignancies worldwide (1). Approximately 25–30% of RCC patients have developed locally advanced or metastatic lesions at the time of initial diagnosis (2, 3). For patients with locally advanced and metastatic renal cancer, although targeted drug therapy has achieved certain efficacy and more clinical trials of drugs are ongoing, the overall prognosis is still poor (4, 5). The preferred treatment for early stage non-metastatic RCC remains radical nephrectomy or partial nephrectomy. Early recurrence or metastasis is still found in 20–30% of patients treated with surgery at follow-up (6). Therefore, the search for better prognostic predictors can be of great help in developing individualized follow-up and treatment plans.

A growing number of studies have confirmed the importance of systemic inflammatory response, local immune response and nutritional status in the progression of malignancy and patient prognosis (7–9). Several blood indicators, including neutrophil-to-lymphocyte ratio (NLR), platelet-to-lymphocyte ratio (PLR) and lymphocyte-to-monocyte ratio (LMR), have been shown correlate with the prognosis of patients with RCC (10, 11). In addition, indicators reflecting nutritional status, such as serum albumin (ALB), hemoglobin and sarcopenia have been identified as postoperative prognostic factors in patients with RCC (12, 13). Jafri et al. (14) developed the advanced lung cancer inflammation index (ALI) to assess the degree of systemic nutrition and inflammation in patients with metastatic non-small cell lung cancer (NSCLC). The ALI combines body mass index (BMI, kg/m^2^), serum ALB (g/dL) and NLR and is defined as BMI × ALB/NLR. In addition, Kim et al. (15) replaced BMI with L3 muscle index (cm^2^/m^2^) to construct a modified ALI (mALI) score and found that low mALI was an independent prognostic risk factor for shorter overall survival (OS).

In this study, we aimed to assess the prognostic value of ALI and mALI on OS in patients undergoing laparoscopic nephrectomy in a multicenter clinical study.

## Materials and Methods

### Study Design and Patients

In this multi-institutional study, we collected 590 patients with RCC who underwent partial or radical nephrectomy between January 2014 and December 2019 at the Department of Urology, Zhongda Hospital Southeast University, the Department of Urology, Shanghai Tenth People's Hospital, and the Department of Urology, Shidong Hospital. All patients were operated by the most experienced urologists in that hospital. Exclusion criteria were as follows: patients combined with other malignancies; patients received other anticancer treatments before surgery; patients lacked complete medical records or were lost to follow-up; patients lacked preoperative laboratory test data. We excluded 150 patients, resulting in 440 patients included in the final study. The methodology of this study followed the criteria in the Declaration of Helsinki (revised in 2013) and received ethical approval from the Ethics Committee of Zhongda Hospital Southeast University (ZDKYSB077) and Ethics Committee of Shanghai Tenth People's Hospital of Tongji University (SHSY-IEC-BG/02.04/04.0-81602469).

### Clinical Data Collection and Follow-Up

Clinicopathological features, laboratory test data, and imaging results for all patients were available from the electronic medical record. Laboratory test data were measured 2 days prior to surgery or closest to the time of surgery, and laboratory data included serum ALB (g/dL), neutrophils, and lymphocytes. The L3 muscle index (cm^2^/m^2^) in the imaging results was determined based on our previous study (13). In addition, we included gender, age, BMI [weight (kg)/height^2^ (m^2^)], cardiovascular disease, diabetes, hypertension, smoking, type of surgery, laterality, AJCC stage, T stage, N stage, M stage, and Fuhrman grade. OS was calculated from the date of surgical treatment to the date of last follow-up or death.

### Statistical Analysis

Categorical variables were expressed as numbers and percentages and analyzed with chi-square tests. As previously described, ALI = BMI × ALB/NLR, and mALI = L3 muscle index × ALB/NLR. Optimal cut-off values for ALI and mALI were determined using X-tile software (version 3.6.1). Kaplan-Meier curves were used to assess the effect of ALI and mALI on OS. Receiver operating characteristic (ROC) curves were used to compare the effect of ALI and mALI's predictive ability on OS and was calculated using the area under the curve (AUC). Univariate and multivariate Cox regression were used to assess the relationship between ALI, mALI and OS, and the associated adjusted hazard ratio (aHR) and 95% confidence interval (CI) were calculated. In multivariate Cox regression analysis, three models were constructed to further assess the relationship between ALI, mALI and OS. Base model: adjusted for age, gender, BMI, hypertension, cardiovascular diseases, diabetes and smoking; core model: base model variables plus surgery type and laterality; extended model: core model variables plus AJCC stage, T stage, N stage, M stage and Fuhrman grade.

Based on the optimal cut-off value of mALI determined by the X-tile software, we divided the patients into a high mALI group (*n* = 216) and a low mALI group (*n* = 214). Considering the differences in some variables between the two groups, we used the “Matching” package in R software to perform 1:1 propensity score matching (PSM) for the high mALI and low mALI groups, adjusting for gender, age, BMI, cardiovascular disease, diabetes, hypertension, smoking, surgery type, laterality, AJCC stage, T stage, N stage, M stage, and Fuhrman grade to further explore the effect of mALI on OS in patients with RCC. Statistical analyses were performed using SPSS software (version 26.0), Graphpad Prism (version 8.3.0), and R software (version 3.6.2). *P* value < 0.05 was considered statistically significant.

## Results

**Figure 1:**
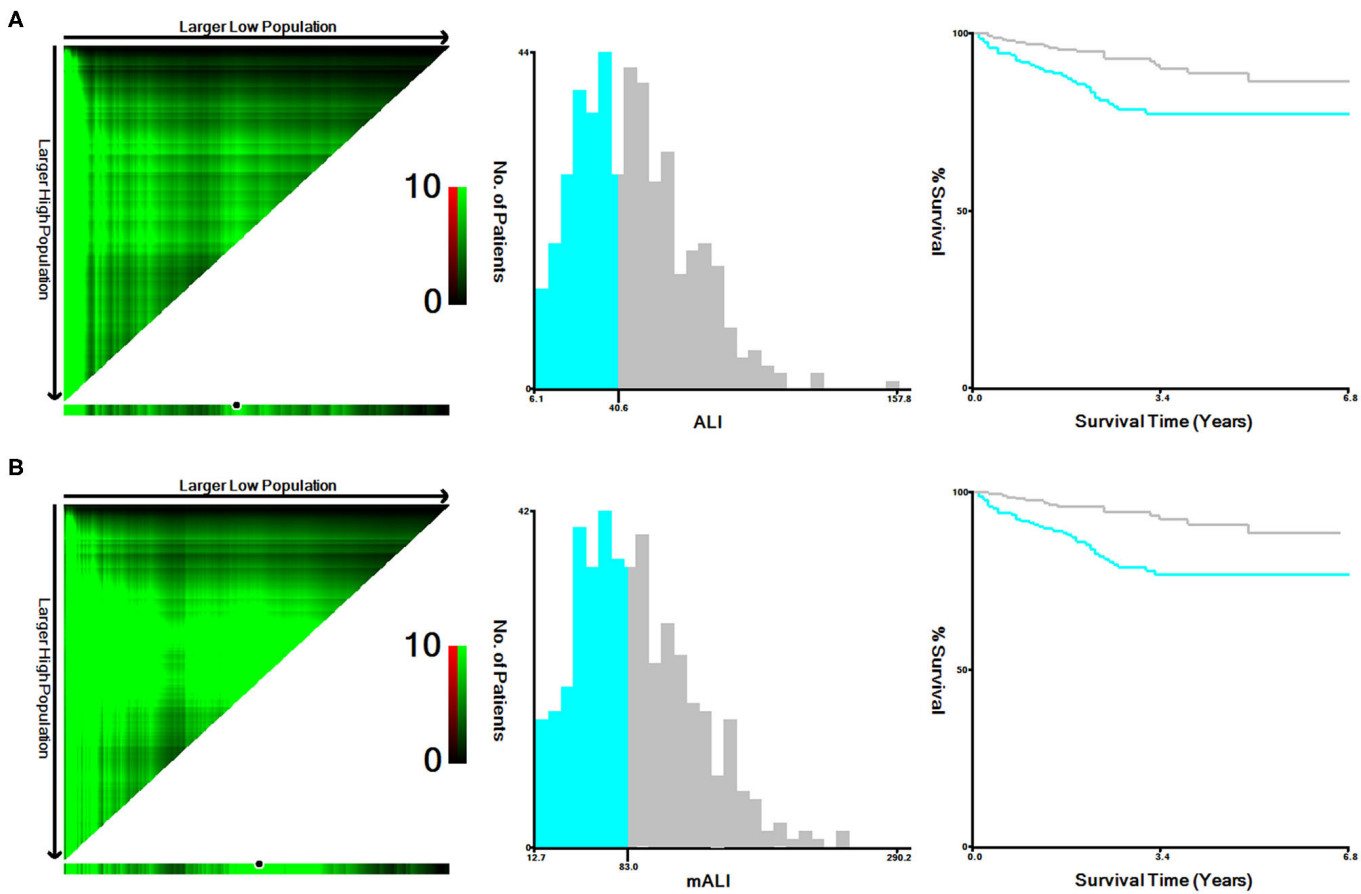
X-tile stratified analysis of ALI and mALI. **(A)** ALI; **(B)** mALI. ALI, advanced lung cancer inflammation index; mALI, modified advanced lung cancer inflammation index.

**Figure 2:**
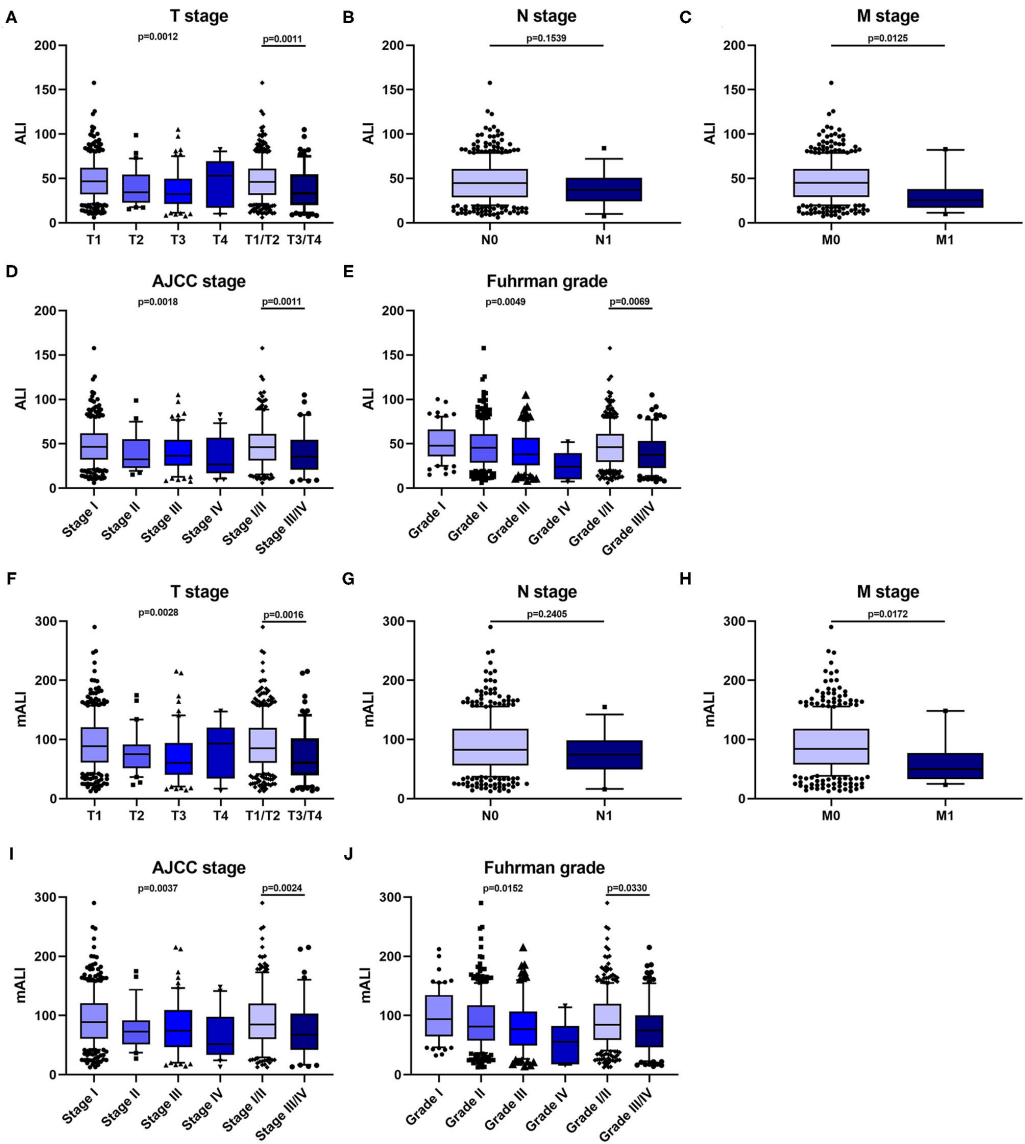
Relationship between patients' clinical characteristics and ALI, mALI. **(A–E)** Levels of ALI in different T-stage **(A)**, N-stage **(B)**, M-stage **(C)**, AJCC stage **(D)**, and Fuhrman grade **(E)**. **(F–J)** Levels of ALI in different T-stage **(F)**, N-stage **(G)**, M-stage **(H)**, AJCC stage **(I)**, and Fuhrman grade **(J)**. ALI, advanced lung cancer inflammation index; mALI, modified advanced lung cancer inflammation index.

According to the X-tile software, the optimal cut-off values for ALI and mALI were 40.6 and 83.0, respectively (Figure 1). Based on the cut-off values, we divided the patients into high ALI and low ALI groups, and high mALI and low mALI groups. The clinicopathological characteristics of all patients were shown in Table 1. Chi-square testing showed that ALI was associated with BMI, hypertension, surgery type, AJCC stage, T stage, M stage and Fuhrman grade, whereas mALI was statistically associated with BMI, surgery type, AJCC stage, T stage, M stage and Fuhrman grade. A higher proportion of patients with BMI ≥ 25kg/m^2^, underwent partial nephrectomy, AJCC I/II stage, T1/T2 stage, M0 stage, and Fuhrman I/II grade were in the high ALI and high mALI groups compared with the low ALI and low mALI groups. In addition, we found that higher T stage, M stage, AJCC stage and Fuhrman grade were associated with lower ALI and lower mALI (Figure 2).

**Table 1:** Clinical characteristics of the patients according to ALI and mALI before PSM.

**Characteristic**	**All**	**ALI**		***P* value**	**mALI**		***P* value**
	**patients**	**Low ALI**	**High ALI**		**Low ALI**	**High ALI**	
	***N* = 440**	***N* = 196**	***N* = 244**		***N* = 224**	***N* = 216**	
Age categorized, y				0.964			0.613
≤ 65	317 (72.0)	141 (71.9)	176 (72.1)		159 (71.0)	158 (73.1)	
>65	123 (28.0)	55 (28.1)	68 (27.9)		65 (29.0)	58 (26.9)	
Gender				0.994			0.177
Male	294 (66.8)	131 (66.8)	163 (66.8)		143 (63.8)	151 (69.9)	
Female	146 (33.2)	65 (33.2)	81 (33.2)		81 (36.2)	65 (30.1)	
BMI categorized, kg/m^2^				<0.001			<0.001
<25	249 (56.6)	138 (70.4)	111 (45.5)		148 (66.1)	101 (46.8)	
≥25	191 (43.4)	58 (29.6)	133 (54.5)		76 (33.9)	115 (53.2)	
Hypertension				0.039			0.137
No	250 (56.8)	122 (62.2)	128 (52.5)		125 (60.3)	115 (53.2)	
Yes	190 (43.2)	74 (37.8)	116 (47.5)		89 (39.7)	101 (46.8)	
Diabetes				0.567			0.924
No	370 (84.1)	167 (85.2)	203 (83.2)		188 (83.9)	182 (84.3)	
Yes	70 (15.9)	29 (14.8)	41 (16.8)		36 (16.1)	34 (15.7)	
Cardiovascular diseases				0.933			0.758
No	389 (88.4)	173 (88.3)	216 (88.5)		197 (87.9)	192 (88.9)	
Yes	51 (11.6)	23 (11.7)	28 (11.5)		27 (12.1)	24 (11.1)	
Smoking				0.369			0.467
No	367 (83.4)	160 (81.6)	207 (84.8)		184 (82.1)	183 (84.7)	
Yes	73 (16.6)	36 (18.4)	37 (15.2)		40 (17.9)	33 (15.3)	
Surgery type				<0.001			<0.001
Partial nephrectomy	266 (60.5)	90 (45.9)	176 (72.1)		105 (46.9)	161 (74.5)	
Radical nephrectomy	174 (39.5)	106 (54.1)	68 (27.9)		119 (53.1)	55 (25.5)	
Laterality				0.405			0.151
Right	217 (49.3)	101 (51.5)	116 (47.5)		118 (52.7)	99 (45.8)	
Left	223 (50.7)	95 (48.5)	128 (52.5)		106 (47.3)	117 (54.2)	
AJCC stage				<0.001			0.003
I	328 (74.5)	128 (65.3)	200 (82.0)		151 (67.4)	177 (81.9)	
II	26 (5.9)	17 (8.7)	9 (3.7)		19 (8.5)	7 (3.2)	
III	61 (13.9)	33 (16.8)	28 (11.5)		36 (16.1)	25 (11.6)	
IV	25 (5.7)	18 (9.2)	7 (2.9)		18 (8.0)	7 (3.2)	
T-stage				<0.001			0.001
T1	335 (76.1)	131 (66.8)	204 (83.6)		154 (68.8)	181 (83.8)	
T2	30 (6.8)	19 (9.7)	11 (9.4)		21 (9.4)	9 (4.2)	
T3	64 (14.5)	41 (20.9)	23 (9.4)		44 (19.6)	20 (9.3)	
T4	11 (2.5)	5 (2.6)	6 (2.5)		5 (2.2)	6 (2.8)	
N-stage				0.227			0.506
N0	423 (96.1)	186 (94.9)	237 (97.1)		214 (95.5)	209 (96.8)	
N1	17 (3.9)	10 (5.1)	7 (2.9)		10 (4.5)	7 (3.2)	
M-stage				<0.001			0.003
M0	421 (95.7)	180 (91.8)	241 (98.8)		208 (92.9)	213 (98.6)	
M1	19 (4.3)	16 (8.2)	3 (1.2)		16 (7.1)	3 (1.4)	
Fuhrman grade				0.017			0.020
I	74 (16.8)	27 (13.8)	47 (19.3)		28 (12.5)	46 (21.3)	
II	274 (62.3)	117 (59.7)	157 (64.3)		141 (62.9)	133 (61.6)	
III	82 (18.6)	44 (22.4)	38 (15.6)		47 (21.0)	35 (16.2)	
IV	10 (2.3)	8 (4.1)	2 (0.8)		8 (3.6)	2 (0.9)	
Urea nitrogen (mean, SD)	6.46, 4.45	6.77, 3.43	6.21, 5.12	0.190	6.62, 3.31	6.29, 5.38	0.433
Creatinine (mean, SD)	112.28, 88.72	123.55, 90.12	103.29, 86.73	0.018	119.82, 85.72	104.50, 91.27	0.071
Uric acid (mean, SD)	277.54, 102.81	281.14, 106.17	274.69, 100.19	0.517	277.33, 107.74	277.76, 97.73	0.965

*PSM, propensity score matching; ALI, advanced lung cancer inflammation index; mALI, modified advanced lung cancer inflammation index; BMI, body mass index; AJCC, american joint committee on cancer; SD, standard deviation*.

**Figure 3:**
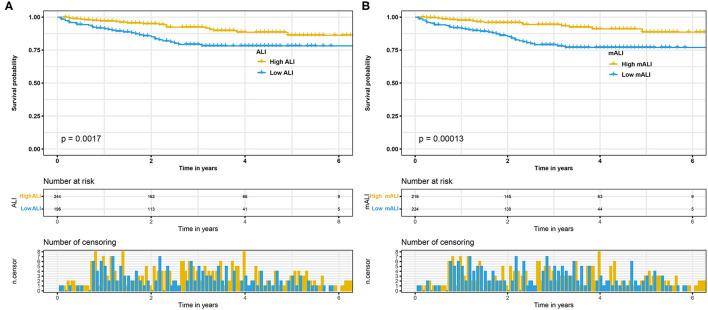
Kaplan-Meier curves for OS stratified by ALI and mALI before PSM. **(A)** ALI OS; **(B)** mALI OS. OS, overall survival; ALI, advanced lung cancer inflammation index; mALI, modified advanced lung cancer inflammation index; PSM, propensity score matching.

We performed survival analysis for the high ALI and low ALI groups, as well as for the high mALI and low mALI groups. Kaplan-Meier curves showed that low ALI (*p* = 0.002) and low mALI (*p* < 0.001) were associated with worse prognosis (Figure 3). Subsequently, we used ROC curves to assess the predictive ability of ALI and mALI for OS. We found that mALI had a better ability to predict OS than ALI (Supplementary Figure 1). In addition, univariate Cox regression analysis showed that low ALI and low mALI were associated with poorer OS (Table 2). Multivariate Cox regression analysis showed that mALI was consistently an independent risk factor for OS, whether in the basic model (low mALI vs. high mALI: aHR = 3.09; 95% CI 1.68–5.69, *p* < 0.001), core model (low mALI vs. high mALI: aHR = 2.20; 95% CI 1.17–4.14, *p* = 0.014) or extended model (low mALI vs. high mALI: aHR = 2.22; 95% CI 1.19–4.13, *p* = 0.012), while ALI was statistically significant only in the basic model (Table 2).

**Table 2:** Relative risk of overall survival (OS) was calculated according to ALI and mALI ^a^.

**Characteristic**	**Univariate analysis**		**Basic model**		**Core model**		**Extended model**	
	**aHR (95% CI)**	***P*-value**	**aHR (95% CI)**	***P*-value**	**aHR (95% CI)**	***P*-value**	**aHR (95% CI)**	***P*-value**
**BEFORE PSM**								
**ALI**								
High	Reference		Reference		Reference		Reference	
Low	2.36 (1.36–4.10)	0.002	2.22 (1.25–3.93)	0.007	1.62 (0.89–2.96)	0.117	1.40 (0.73–2.66)	0.309
**mALI**								
High	Reference		Reference		Reference		Reference	
Low	3.09 (1.68–5.68)	<0.001	3.09 (1.68–5.69)	<0.001	2.20 (1.17–4.14)	0.014	2.22 (1.19–4.13)	0.012
**AFTER PSM**								
**mALI**								
High	Reference		Reference		Reference		Reference	
Low	2.16 (1.04–4.48)	0.039	2.26 (1.09–4.70)	0.029	2.26 (1.08–4.70)	0.030	2.88 (1.33–6.26)	0.007

a *Adjusted covariates: Basic model: age, gender, BMI, hypertension, diabetes, cardiovascular diseases, and smoking; Core model: basic model plus surgery type and laterality; Extended model: core model plus AJCC stage, T stage, N stage, M stage, and fuhrman grade. PSM, propensity score matching; BMI, body mass index; AJCC, american joint committee on cancer; aHR, adjusted hazard ratio; CI, confidence interval; mALI, modified advanced lung cancer inflammation index*.

**Figure 4:**
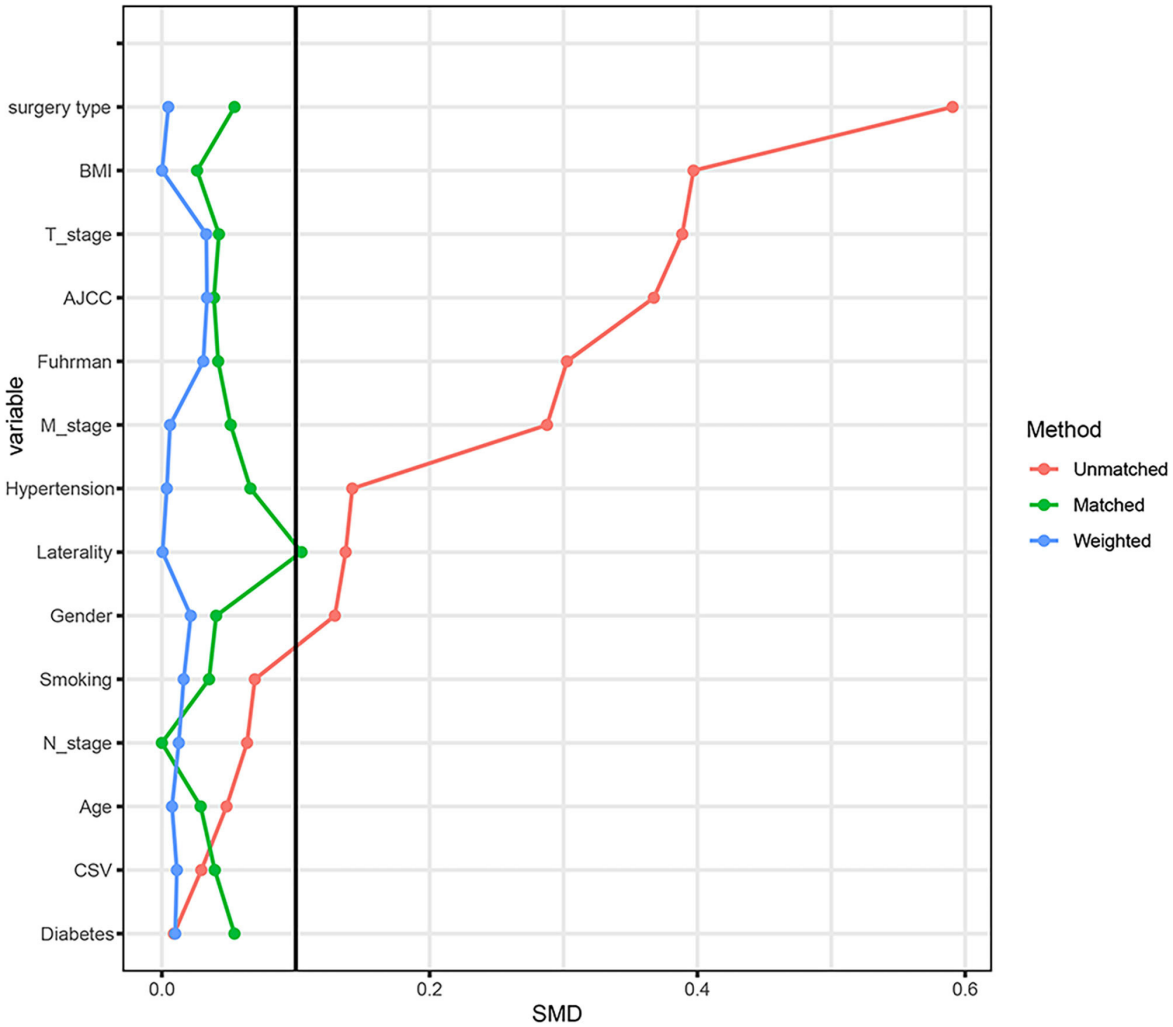
The standardized mean difference (SMD) results of different variables after PSM. PSM, propensity score matching.

**Figure 5:**
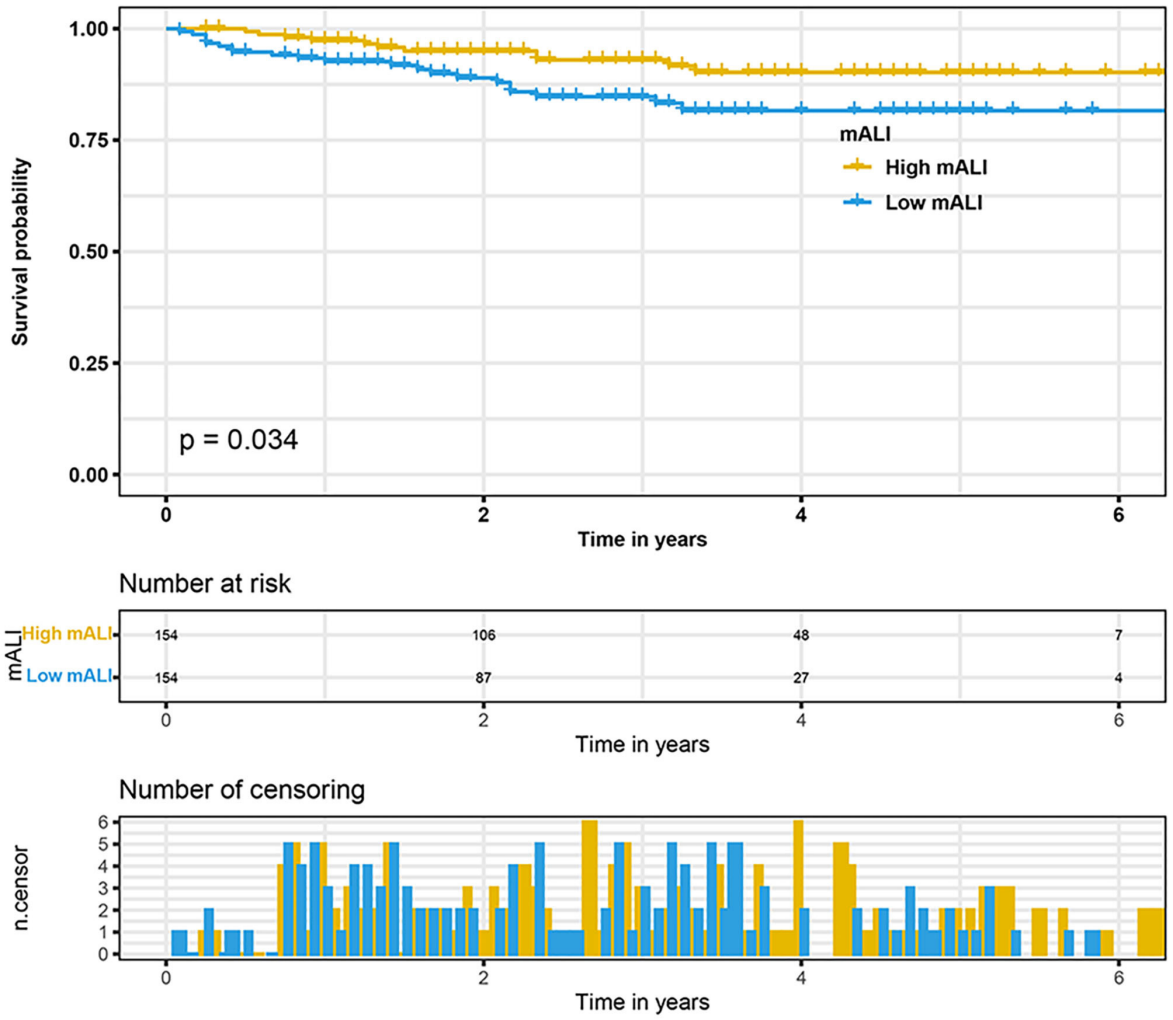
Kaplan-Meier curves for OS stratified by mALI after PSM. OS, overall survival; mALI, modified advanced lung cancer inflammation index; PSM, propensity score matching.

Considering the effect of other confounding variables, we performed a 1:1 PSM analysis for the high mALI and low mALI groups and adjusted for the 14 variables of gender, age, BMI, cardiovascular disease, diabetes, hypertension, smoking, surgery type, laterality, AJCC stage, T stage, N stage, M stage, and Fuhrman grade (Figure 4). After the PSM analysis, 154 patients were included in the high mALI and low mALI groups, respectively. Clinicopathological characteristics of 308 patients after PSM were shown in Supplementary Table 1. We performed survival analysis in 308 patients and Kaplan-Meier curves still showed that low mALI (*p* = 0.034) was associated with a poorer prognosis (Figure 5). Univariate and multivariate Cox regression analyses showed that low mALI was associated with a higher risk and that low mALI was associated with a 188% higher risk compared to high mALI in the extended model (aHR = 2.88; 95% CI 1.33–6.26, *p* = 0.007) (Table 2).

## Discussion

Our study is the first study assessing the prognostic value of ALI and mALI in RCC. In this multi-institutional retrospective study, we included clinical data from 440 patients who underwent nephrectomy and used Kaplan-Meier curves, ROC curves, and univariate and multivariate Cox regression analyses to explore the correlation between ALI, mALI, and OS. We found that low ALI and low mALI were correlated with poor prognosis, mALI was an independent risk factor for OS, and mALI was a better predictor of OS in RCC patients than ALI. In addition, we further performed 1:1 PSM on patients in the low mALI and high mALI groups and found that mALI was still an independent risk factor for OS.

L3 muscle index is a common indicator of sarcopenia. Sarcopenia is an age-related syndrome and is considered an emerging indicator that can reflect nutritional status (16). Sarcopenia is defined as an age-related syndrome of reduced skeletal muscle mass, decreased muscle strength and/or decreased physical performance (17). Currently, sarcopenia can be assessed by measuring the L3 lumbar skeletal muscle index (SMI) (18). In recent years, there were increasing evidences that oncology patients often had comorbid sarcopenia. The prevalence of sarcopenia ranged from 20 to 70% in different tumors (19). Studies have shown that as SMI decreases, oncology patients have a poorer prognosis and an increased risk of complications (20). Our previous studies found that sarcopenia was a risk factor for survival time in patients with bladder cancer and RCC (13, 21). Sarcopenia played an important role in the treatment prognosis of oncology patients, and nutritional, exercise and pharmacological interventions for patients with sarcopenia could reduce the occurrence of post-treatment complications and improve the prognosis of patients with oncology (22).

ALB is a product synthesized by the liver and is an important component of human serum protein, which has an important role in the transport and synthesis of substances in the organism. Serum ALB is a common marker used to assess the nutritional status of patients, and low serum ALB level indicates that the patient is malnourished (23). In addition to being an indicator of nutritional status, serum ALB may also be associated with mechanisms of inflammatory response (24). Studies have demonstrated that preoperative low serum ALB levels may be considered as a marker of systemic inflammation and a poor prognostic indicator of survival outcome in cancer patients (25, 26).

Many studies have shown that the development of malignant tumors is closely related to the tumor microenvironment (27). Inflammatory cells, such as neutrophils, lymphocytes, and monocytes, are important components of the tumor microenvironment, and their mediated inflammatory responses can promote tumor cell proliferation, invasion, metastasis, and immune escape (7, 28). The combination of multiple inflammatory cells, such as NLR, PLR and LMR, has been shown to correlate with the prognosis of various cancers (29, 30). NLR is an evaluation indicator reflecting the systemic inflammatory response and is one of the earliest and most classical inflammatory indicators found. The literature reports that preoperative NLR levels are significantly associated with postoperative tumor survival in a variety of solid tumors (31).

In order to better assess patient prognosis, Jafri et al. (14) developed an index (ALI) that could reflect the degree of systemic nutrition and inflammation in patients based on three indicators: BMI, ALB and NLR, and found that low ALI was a poor prognostic indicator for patients with advanced NSCLC. In addition, subsequent studies have shown that low ALI can be used to assess the prognosis of various malignancies, such as small cell lung cancer (SCLC), colorectal cancer and pancreatic carcinoma (32, 33). Considering that BMI cannot directly measure body fat and skeletal muscle content, Kim et al. (15) replaced BMI with L3 muscle index to construct a modified ALI (mALI) score and found that low mALI was an independent prognostic risk factor for SCLC patients OS shortening. In the present study, we compared the predictive ability of ALI and mALI for OS, and found that mALI better predicted OS in RCC patients and that mALI was an independent risk factor for OS.

Despite the positive results obtained in this study, there are several limitations to this study. First, although this study was a three-institution multicenter study, it was still a retrospective study and required an expanded sample for prospective studies. Second, we did not assess patients' quality of life or postoperative nutritional status. Final, we did not include other treatments in the study, which may also have an impact on prognosis.

## Conclusion

In general, we found that both ALI and mALI were associated with poor prognosis in patients with RCC, but mALI was a better predictor of OS than ALI, and mALI was an independent prognostic factor for OS in patients with RCC undergoing laparoscopic nephrectomy.

## Data Availability Statement

The raw data supporting the conclusions of this article will be made available by the authors, without undue reservation.

## Ethics Statement

The studies involving human participants were reviewed and approved by the Ethics Committee of Zhongda Hospital Southeast University (ZDKYSB077) and Ethics Committee of Shanghai Tenth People's Hospital of Tongji University (SHSY-IEC-BG/02.04/04.0-81602469). The patients/participants provided their written informed consent to participate in this study.

## Author Contributions

WM, JG, SC, and MC: conception and design. JG, SC, and MC: administrative support. WM, KW, YW, and JN: Provision of study materials or patients and manuscript writing. WM, HZ, YW, ZW, and RL: collection and assembly of data. WM and KW: data analysis and interpretation. All authors contributed to the article, final approval of manuscript, and approved the submitted version.

## Funding

This study was supported by National Natural Science Foundation of China (No. 82070773), Natural Science Foundation of Jiangsu Province (No. BK20201271), Scientific Research Foundation of Graduate School of Southeast University (No. YBPY2173), Postgraduate Research & Practice Innovation Program of Jiangsu Province (No. KYCX21_0156), Jiangsu Provincial Key Research and Development Program (No. BE2019751), Innovative Team of Jiangsu Provincial (No. 2017ZXKJQW07), and National Key Research and Development Program of China (No. SQ2017YFSF090096).

## Conflict of Interest

The authors declare that the research was conducted in the absence of any commercial or financial relationships that could be construed as a potential conflict of interest.

## Publisher's Note

All claims expressed in this article are solely those of the authors and do not necessarily represent those of their affiliated organizations, or those of the publisher, the editors and the reviewers. Any product that may be evaluated in this article, or claim that may be made by its manufacturer, is not guaranteed or endorsed by the publisher.

## Supplementary Material

Supplementary Figure 1 Comparison of area under ROC curves for ALI and mALI in predicting OS. ROC, receiver operating characteristic; OS, overall survival; ALI, advanced lung cancer inflammation index; mALI, modified advanced lung cancer inflammation index.

## Abbreviations

RCC: renal cell carcinoma
NLR: neutrophil-to-lymphocyte ratio
PLR: platelet-to-lymphocyte ratio
LMR: lymphocyte-to-monocyte ratio
ALB: albumin
ALI: advanced lung cancer inflammatory index
mALI: modified advanced lung cancer inflammatory index
BMI: body mass index
OS: overall survival
ROC: receiver operating characteristic
AUC: area under the curve
aHR: adjusted hazard ratio
CI: confidence interval
PSM: propensity score matching
SMI: skeletal muscle index.

## References

[1] Sung H Ferlay J Siegel RL Laversanne M Soerjomataram I Jemal A. Global cancer statistics 2020: GLOBOCAN estimates of incidence and mortality worldwide for 36 cancers in 185 countries. CA Cancer J Clin. (2021) 71:209–49. 10.3322/caac.21660 33538338

[2] Ljungberg B Bensalah K Canfield S Dabestani S Hofmann F Hora M. EAU guidelines on renal cell carcinoma: 2014 update. Eur Urol. (2015) 67:913–24. 10.1016/j.eururo.2015.01.005 25616710

[3] Mao W Wang K Xu B Zhang H Sun S Hu Q. ciRS-7 is a prognostic biomarker and potential gene therapy target for renal cell carcinoma. Mol Cancer. (2021) 20:142. 10.1186/s12943-021-01443-2 34740354 PMC8570002

[4] Yong C Stewart GD Frezza C. Oncometabolites in renal cancer. Nat Rev Nephrol. (2020) 16:156–72. 10.1038/s41581-019-0210-z 31636445 PMC7030949

[5] Bianchi M Sun M Jeldres C Shariat SF Trinh QD Briganti A. Distribution of metastatic sites in renal cell carcinoma: a population-based analysis. Ann Oncol. (2012) 23:973–80. 10.1093/annonc/mdr362 21890909

[6] Tannir NM Pal SK Atkins MB. Second-line treatment landscape for renal cell carcinoma: a comprehensive review. Oncologist. (2018) 23:540–55. 10.1634/theoncologist.2017-0534 29487224 PMC5947457

[7] Diakos CI Charles KA McMillan DC Clarke SJ. Cancer-related inflammation and treatment effectiveness. Lancet Oncol. (2014) 15:e493–503. 10.1016/S1470-2045(14)70263-3 25281468

[8] Souza Cunha M Wiegert EVM Calixto-Lima L Oliveira LC. Relationship of nutritional status and inflammation with survival in patients with advanced cancer in palliative care. Nutrition. (2018) 51–52:98–103. 10.1016/j.nut.2017.12.004 29625409

[9] De Giorgi U Procopio G Giannarelli D Sabbatini R Bearz A Buti S. Association of systemic inflammation index and body mass index with survival in patients with renal cell cancer treated with nivolumab. Clin Cancer Res. (2019) 25:3839–46. 10.1158/1078-0432.CCR-18-3661 30967420

[10] Semeniuk-Wojtas A Lubas A Stec R Syrylo T Niemczyk S Szczylik C. Neutrophil-to-lymphocyte ratio, platelet-to-lymphocyte ratio, and c-reactive protein as new and simple prognostic factors in patients with metastatic renal cell cancer treated with tyrosine kinase inhibitors: a systemic review and meta-analysis. Clin Genitourin Cancer. (2018) 16:e685–93. 10.1016/j.clgc.2018.01.010 29454639

[11] Gu L Ma X Xie Y Li H Wang L Chen L. Pretreatment lymphocyte to monocyte ratio is an independent prognostic factor in metastatic clear cell renal cell carcinoma. Clin Genitourin Cancer. (2017) 15:e369–77. 10.1016/j.clgc.2016.12.001 28038932

[12] Motzer RJ Bacik J Schwartz LH Reuter V Russo P Marion S. Prognostic factors for survival in previously treated patients with metastatic renal cell carcinoma. J Clin Oncol. (2004) 22:454–63. 10.1200/JCO.2004.06.132 14752067

[13] Mao W Wang K Zhang H Lu H Sun S Tian C. Sarcopenia as a poor prognostic indicator for renal cell carcinoma patients undergoing nephrectomy in China: A multicenter study. Clin Transl Med. (2021) 11:e270. 10.1002/ctm2.270 33463055 PMC7775986

[14] Jafri SH Shi R Mills G. Advance lung cancer inflammation index (ALI) at diagnosis is a prognostic marker in patients with metastatic non-small cell lung cancer (NSCLC): a retrospective review. BMC Cancer. (2013) 13:158. 10.1186/1471-2407-13-158 23530866 PMC3618002

[15] Kim EY Kim N Kim YS Seo JY Park I Ahn HK. Prognostic significance of modified advanced lung cancer inflammation index (ALI) in patients with small cell lung cancer_ comparison with original ALI. PLoS ONE. (2016) 11:e0164056. 10.1371/journal.pone.0164056 27706243 PMC5051688

[16] Wilson D Jackson T Sapey E Lord JM. Frailty and sarcopenia: the potential role of an aged immune system. Ageing Res Rev. (2017) 36:1–10. 10.1016/j.arr.2017.01.006 28223244

[17] Cruz-Jentoft AJ Bahat G Bauer J Boirie Y Bruyere O Cederholm T. Sarcopenia: revised European consensus on definition and diagnosis. Age Ageing. (2019) 48:16–31. 10.1093/ageing/afz046 30312372 PMC6322506

[18] Prado CM Lieffers JR McCargar LJ Reiman T Sawyer MB Martin L. Prevalence and clinical implications of sarcopenic obesity in patients with solid tumours of the respiratory and gastrointestinal tracts: a population-based study. Lancet Oncol. (2008) 9:629–35. 10.1016/S1470-2045(08)70153-0 18539529

[19] Chen LK Liu LK Woo J Assantachai P Auyeung TW Bahyah KS. Sarcopenia in Asia: consensus report of the Asian working group for sarcopenia. J Am Med Dir Assoc. (2014) 15:95–101. 10.1016/j.jamda.2013.11.025 24461239

[20] Shachar SS Williams GR Muss HB Nishijima TF. Prognostic value of sarcopenia in adults with solid tumours: a meta-analysis and systematic review. Eur J Cancer. (2016) 57:58–67. 10.1016/j.ejca.2015.12.030 26882087

[21] Mao W Ma B Wang K Wu J Xu B Geng J. Sarcopenia predicts prognosis of bladder cancer patients after radical cystectomy: a study based on the Chinese population. Clin Transl Med. (2020) 10:e105. 10.1002/ctm2.105 32535994 PMC7403655

[22] Martin L Birdsell L Macdonald N Reiman T Clandinin MT McCargar LJ. Cancer cachexia in the age of obesity: skeletal muscle depletion is a powerful prognostic factor, independent of body mass index. J Clin Oncol. (2013) 31:1539–47. 10.1200/JCO.2012.45.2722 23530101

[23] McMillan DC Watson WS O'Gorman P Preston T Scott HR McArdle CS. Albumin concentrations are primarily determined by the body cell mass and the systemic inflammatory response in cancer patients with weight loss. Nutr Cancer. (2001) 39:210–3. 10.1207/S15327914nc392_8 11759282

[24] Rodoman GV Shalaeva TI Dobretsov GE Naumov EK Obolenskii VN. [Serum albumin in systemic inflammatory reaction syndrome]. Anesteziol Reanimatol. (2006) 62–4. 10.1016/j.jhep.2014.04.012 16758948

[25] Ku JH Kim M Choi WS Kwak C Kim HH. Preoperative serum albumin as a prognostic factor in patients with upper urinary tract urothelial carcinoma. Int Braz J Urol. (2014) 40:753–62. 10.1590/S1677-5538.IBJU.2014.06.06 25615244

[26] Han S Huang Y Li Z Hou H Wu A. The prognostic role of preoperative serum albumin levels in glioblastoma patients. BMC Cancer. (2015) 15:108. 10.1186/s12885-015-1125-0 25880463 PMC4355370

[27] Ji F Liang Y Fu SJ Guo ZY Shu M Shen SL. A novel and accurate predictor of survival for patients with hepatocellular carcinoma after surgical resection: the neutrophil to lymphocyte ratio (NLR) combined with the aspartate aminotransferase/platelet count ratio index (APRI). BMC Cancer. (2016) 16:137. 10.1186/s12885-016-2189-1 26907597 PMC4763424

[28] Gu L Ma X Li H Chen L Xie Y Zhao C. Prognostic value of preoperative inflammatory response biomarkers in patients with sarcomatoid renal cell carcinoma and the establishment of a nomogram. Sci Rep. (2016) 6:23846. 10.1038/srep23846 27035802 PMC4817406

[29] Dupre A Malik HZ. Inflammation and cancer: what a surgical oncologist should know. Eur J Surg Oncol. (2018) 44:566–70. 10.1016/j.ejso.2018.02.209 29530345

[30] Demirdal T Sen P. The significance of neutrophil-lymphocyte ratio, platelet-lymphocyte ratio and lymphocyte-monocyte ratio in predicting peripheral arterial disease, peripheral neuropathy, osteomyelitis and amputation in diabetic foot infection. Diabetes Res Clin Pract. (2018) 144:118–25. 10.1016/j.diabres.2018.08.009 30176260

[31] Mei Z Shi L Wang B Yang J Xiao Z Du P. Prognostic role of pretreatment blood neutrophil-to-lymphocyte ratio in advanced cancer survivors: a systematic review and meta-analysis of 66 cohort studies. Cancer Treat Rev. (2017) 58:1–13. 10.1016/j.ctrv.2017.05.005 28602879

[32] Hua X Chen J Wu Y Sha J Han S Zhu X. Prognostic role of the advanced lung cancer inflammation index in cancer patients: a meta-analysis. World J Surg Oncol. (2019) 17:177. 10.1186/s12957-019-1725-2 31677642 PMC6825711

[33] Topkan E Mertsoylu H Ozdemir Y Sezer A Kucuk A Besen AA. Prognostic usefulness of advanced lung cancer inflammation index in locally-advanced pancreatic carcinoma patients treated with radical chemoradiotherapy. Cancer Manag Res. (2019) 11:8807–15. 10.2147/CMAR.S222297 31632140 PMC6789411

