# Supplementary material for: Prognostic Significance of Modified Advanced Lung Cancer Inflammation Index in Patients With Renal Cell Carcinoma Undergoing Laparoscopic Nephrectomy: A Multi-Institutional, Propensity Score Matching Cohort Study
Source: Front Nutr. 2022 Jan 20;8:781647. doi: 10.3389/fnut.2021.781647 (PMC8811296; doi:10.3389/fnut.2021.781647)
Supplement: Supplementary file 1 [file Table_1.docx]

**Table** **S1.** Clinical characteristics of the patients according to mALI after PSM.

| **Characteristic** | **All** | **mALI** | | **P**  **value** |
| --- | --- | --- | --- | --- |
|  | **patients** | **Low ALI** | **High ALI** |  |
|  | **N=308** | **N=154** | **N=154** |  |
| Age categorized, y |  |  |  | 0.799 |
| ≤ 65 | 222 (72.1) | 112 (72.7) | 110 (71.4) |  |
| > 65 | 86 (27.9) | 42 (27.3) | 44 (28.6) |  |
| Gender |  |  |  | 0.722 |
| Male | 197 (64.0) | 100 (64.9) | 97 (63.0) |  |
| Female | 111 (36.0) | 54 (35.1) | 57 (37.0) |  |
| BMI categorized, kg/m^2^ |  |  |  | 0.818 |
| < 25 | 178 (57.8) | 88 (57.1) | 90 (58.4) |  |
| ≥ 25 | 130 (42.2) | 66 (42.9) | 64 (41.6) |  |
| Hypertension |  |  |  | 0.562 |
| No | 183 (59.4) | 89 (57.8) | 94 (61.0) |  |
| Yes | 125 (40.6) | 65 (42.4) | 60 (39.0) |  |
| Diabetes |  |  |  | 0.635 |
| No | 261 (84.7) | 129 (83.8) | 132 (85.7) |  |
| Yes | 47 (15.3) | 25 (16.2) | 22 (14.3) |  |
| Cardiovascular diseases |  |  |  | 0.729 |
| No | 270 (87.7) | 134 (87.0) | 136 (88.3) |  |
| Yes | 38 (12.3) | 20 (13.0) | 18 (11.7) |  |
| Smoking |  |  |  | 0.757 |
| No | 258 (83.8) | 128 (83.1) | 130 (84.4) |  |
| Yes | 50 (16.2) | 26 (16.9) | 24 (15.6) |  |
| Surgery type |  |  |  | 0.634 |
| Partial nephrectomy | 198 (64.3) | 97 (63.0) | 101 (65.6) |  |
| Radical nephrectomy | 110 (35.7) | 57 (37.0) | 53 (34.4) |  |
| Laterality |  |  |  | 0.361 |
| Right | 164 (53.2) | 78 (50.6) | 86 (55.8) |  |
| Left | 144 (46.8) | 76 (49.4) | 68 (44.2) |  |
| AJCC stage |  |  |  | 0.990 |
| I | 244 (79.2) | 122 (79.2) | 122 (79.2) |  |
| II | 14 (4.5) | 7 (4.5) | 7 (4.5) |  |
| III | 39 (12.7) | 19 (12.3) | 20 (13.0) |  |
| IV | 11 (3.6) | 6 (3.9) | 5 (3.2) |  |
| T-stage |  |  |  | 0.987 |
| T1 | 248 (80.5) | 124 (80.5) | 124 (80.5) |  |
| T2 | 16 (5.2) | 8 (5.2) | 8 (5.2) |  |
| T3 | 35 (11.4) | 17 (11.0) | 18 (11.7) |  |
| T4 | 9 (2.9) | 5 (3.2) | 4 (2.6) |  |
| N-stage |  |  |  | 1.00 |
| N0 | 300 (97.4) | 150 (97.4) | 150 (97.4) |  |
| N1 | 8 (2.6) | 4 (2.6) | 4 (2.6) |  |
| M-stage |  |  |  | 0.652 |
| M0 | 303 (98.4) | 152 (98.7) | 151 (98.1) |  |
| M1 | 5 (1.6) | 2 (1.3) | 3 (1.9) |  |
| Fuhrman grade |  |  |  | 0.987 |
| I | 56 (18.2) | 27 (17.5) | 29 (18.8) |  |
| II | 191 (62.0) | 97 (63.0) | 94 (61.0) |  |
| III | 57 (18.5) | 28 (18.2) | 29 (18.8) |  |
| IV | 4 (1.3) | 2 (1.3) | 2 (1.3) |  |
| Urea nitrogen (mean, SD) | 6.33, 4.84 | 6.21, 2.85 | 6.45, 6.23 | 0.662 |
| Creatinine (mean, SD) | 108.54, 89.19 | 109.02, 69.13 | 108.06, 105.65 | 0.925 |
| Uric acid (mean, SD) | 270.88, 101.67 | 271.14, 105.40 | 270.63, 98.22 | 0.965 |

**Abbreviations:**

PSM, propensity score matching; mALI, modified advanced lung cancer inflammation index; BMI, Body mass index; AJCC, American Joint Committee on Cancer; SD, standard deviation.
