# Supplementary figures and images for: Prognostic Significance of Modified Advanced Lung Cancer Inflammation Index in Patients With Renal Cell Carcinoma Undergoing Laparoscopic Nephrectomy: A Multi-Institutional, Propensity Score Matching Cohort Study
Source: Front Nutr. 2022 Jan 20;8:781647. doi: 10.3389/fnut.2021.781647 (PMC8811296; doi:10.3389/fnut.2021.781647)

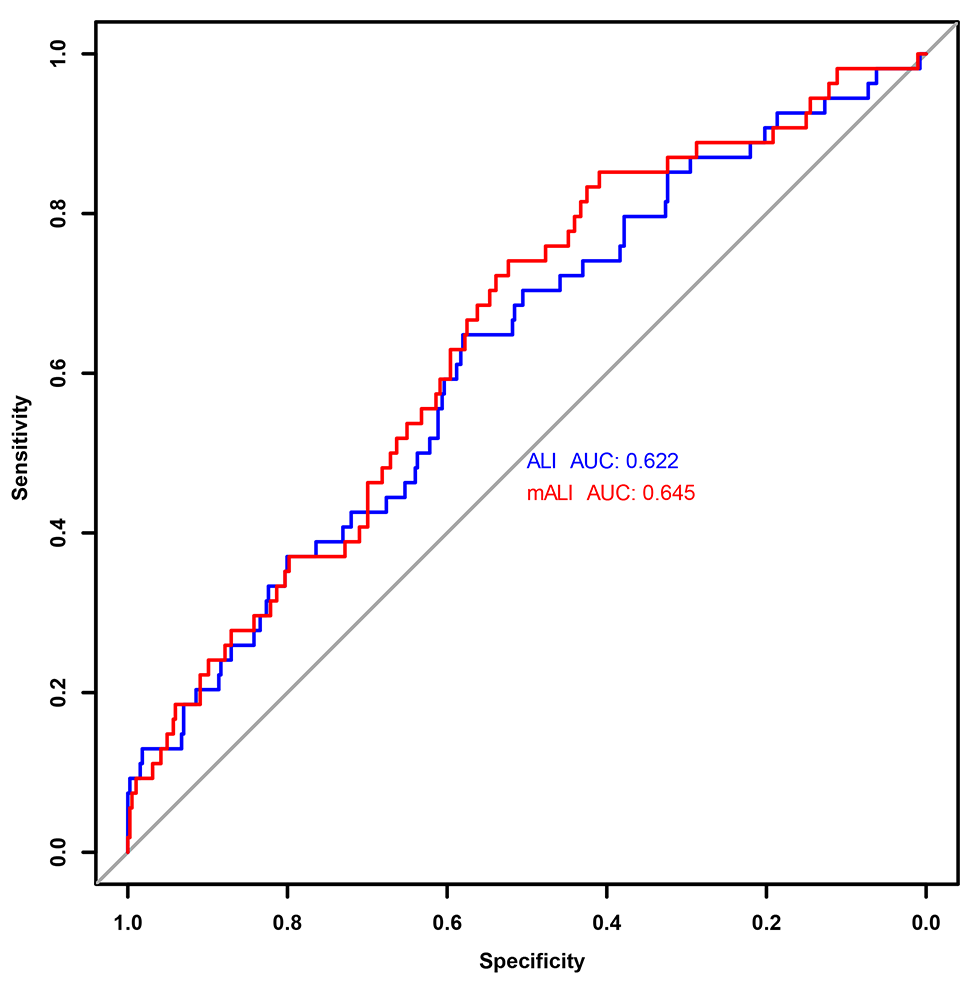

Supplement: Supplementary Figure 1 — Comparison of area under ROC curves for ALI and mALI in predicting OS. ROC, receiver operating characteristic; OS, overall survival; ALI, advanced lung cancer inflammation index; mALI, modified advanced lung cancer inflammation index. [file Image_1.TIF]
